# Stopover departure decisions in spring: pre-Saharan migrants stay longer and are more selective for favourable wind than trans-Saharan migrants

**DOI:** 10.1186/s40462-025-00575-0

**Published:** 2025-09-22

**Authors:** Thomas Klinner, Thiemo Karwinkel, Florian Packmor, Heiko Schmaljohann

**Affiliations:** 1 Institute of Avian Research “Vogelwarte Helgoland”, An der Vogelwarte 21, 26386 Wilhelmshaven, Germany; 2 Naturwacht Brandenburg, Heinrich-Mann-Allee 18/19, 14473 Potsdam, Germany; 3 School of Mathematics and Science, Institute of Biology and Environmental Sciences, Carl von Ossietzky Universität Oldenburg, Ammerländer Heerstraße 114–118, 26129 Oldenburg, Germany; 4 Lower Saxon Wadden Sea National Park Authority, Virchowstraße 1, 26382 Wilhelmshaven, Germany

**Keywords:** Departure decision, trans-Saharan migrants, pre-Saharan migrants, Songbird, Radio-tracking, Weather conditions, Energy stores, Departure direction

## Abstract

**Background:**

Birds that breed in Europe and winter south of the Sahara, so-called trans-Saharan migrants, generally migrate longer distances than pre-Saharan migrants. The latter are expected to be less time constrained during autumn migration than the former. As such, pre-Saharan migrants are assumed to be more selective for favourable weather conditions and are more likely to minimise energy cost of migration than trans-Saharan migrants. While this pattern is supported for autumn migration, it is less well understood for spring migration. Since the optimal arrival timing at the breeding areas is generally under selection pressure to arrive ‘early’, i.e. before ‘competitors’, and since this advantage is likely to hold across migration strategies, we predict that the general differences in decision making between pre- and trans-Saharan migrants will also be manifested during spring migration.

**Methods:**

We radio-tracked three trans-Saharan (Common Redstart *Phoenicurus phoenicurus*, Garden Warbler *Sylvia borin* and Northern Wheatear *Oenanthe oenanthe*) and two pre-Saharan (European Robin *Erithacus rubecula* and Dunnock *Prunella modularis*) migrants during stopover using a regional network of Motus receiving stations. We analysed the night-to-night and within-night departure decisions in relation to weather and energy stores, and compared species’ departure direction with the location of their ring recoveries.

**Results:**

Trans-Saharan migrants stopped-over shorter and were less selective for favourable wind conditions than pre-Saharan migrants. The positive effect of high energy stores and low cloud cover on departure probability was a consistent pattern. Within-night departure times did not differ between migration strategies. Departure directions were in line with geographical mean location of ring recoveries for Common Redstart, European Robin and Dunnock.

**Conclusions:**

Our results suggest that pre-Saharan migrants are less time-constrained and follow an energy-saving strategy more strongly than trans-Saharan migrants that seem to have a stronger urge to migrate fast in spring. Since a similar pattern exists for autumn migration, we suggest that how the species-specific migration strategies and associated time constraints affect stopover decision making in both migration seasons is a general mechanism in migratory songbirds.

**Supplementary Information:**

The online version contains supplementary material available at 10.1186/s40462-025-00575-0.

## Background

Migrant birds track temporally and spatially limited resources, e.g., food, safety and potential mates, through their seasonal movements [1]. Especially in night-time migratory songbirds, an innate migration program provides the spatial and temporal schedules of migration and the framework within which extrinsic (e.g., wind) and intrinsic (e.g., energy stores) factors modify endogenously controlled migratory traits [2]. As such, innate migration programs specify the general migration distance and timing of migration, departure from stopover and the assessment of and response to extrinsic and intrinsic factors during migration [3].

We generally categorise obligate migrants as long-, medium- or short-distance migrants, on the basis of their migration distance, although the distances travelled can vary considerably within and between species, populations and even the categories [4]. Within the Western Palaearctic-African migration system, the yes/no-seasonal passage of the Sahara Desert provides an additional classification criterion to group birds into so-called trans- or pre-Saharan migrants. This classification may be more biologically meaningful than migration distance alone, because the evolutionary processes acting on trans-Sahara migrants as they cross the Mediterranean Sea, the Saharan Desert and migrate long distances, jointly result in their specific adaptations in comparison to pre-Saharan migrants. Those adaptations include: life-history strategies, e.g., fewer, smaller clutches [5]; specific behaviour, e.g., more pronounced nocturnal migration [6]; higher airspeed [7–10]; aerodynamics/morphology, e.g., lower lean body mass [11] or more pointed wings [12].

Optimal migration theory predicts that for trans-Saharan migrants, the specific time constraints associated with the urge to negotiate the Sahara Desert and to travel fast over long distances should result in higher migration speeds, even at the expense of increased energetic costs – the so-called time-minimising strategy [13–15]. In contrast to maximising the speed of migration, minimising energy expenditure during ‘barrier-free’ and short migrations appears to be more advantageous for pre-Saharan migrants – the so-called energy-minimising strategy [16, 17].

Next to the distance travelled, migration theory also predicts that spring migration schedules are additionally influenced by the energetic strategies for arrival at the breeding area: Either birds speed up migration by the cost of arriving at the breeding area with depleted energy stores; or alternatively, birds migrate more energy-efficient, i.e. slower, arrive later, and probably with higher energy reserves than under faster migration [18, 19]. Early arrival under the time minimising strategy secures the ‘most favourable’ breeding territories and ‘high’ mating opportunities [20, 21]. The available energy stores under the ‘slow’ energy minimising strategy may secure survival at the breeding area in case of unfavourable environmental conditions upon arrival, and ‘early’ egg production, e.g. for capital breeding [22]. In songbirds, independent of being pre- and trans-Saharan migrant, the former advantage seems to be ecologically more important than the latter, which is probably more relevant in other bird groups, like shorebirds [23, 24] and geese [25]. In those groups also capital breeding plays a more important role than in smaller songbirds [22, 26].

Selection pressures for either fast or energy-efficient migration has resulted in specific adaptations in songbirds and probably shaped the decision-making process regarding when to interrupt migratory endurance flights or resume migration after a stopover [3]. The latter is the period between flights when individuals rest, recover and refuel [3]. In line with the abovementioned predictions, trans-Saharan migrants during autumn migration have shown higher migration speeds [8, 9, 27, 28], shorter stopovers [17, 29] and earlier departures within the night than pre-Saharan migrants [17]. Trans-Saharan migrants are also less selective for favourable wind conditions in-flight [30] and at stopovers when resuming migration [17]. It was, therefore suggested that the selectivity of pre-Saharan migrants minimises energy cost of transport.

To the best of our knowledge, only Rüppel et al. [29] assessed whether departure decisions are also migration-strategy specific during spring migration. Although they found that the day-to-day departure probability was higher for trans- than for pre-Saharan migrants, as during autumn migration [17], the species of both strategies did not differ in their departure behaviour in response to meteorological conditions. An “early”, i.e. optimal, arrival in the breeding area is strongly correlated with high reproductive success [20, 21], while a late arrival time in the wintering grounds is less associated with increased fitness costs than in spring [31]. Thus, the urge to migrate “fast” should generally, i.e. independent of migration strategy, be higher in spring than in autumn [30, 32]. Selection on migratory decisions and behaviour of birds with different migration strategies could, therefore, potentially be more similar in spring than in autumn, but support for this is currently scarce [29]. The evolutionary advantage of fast migration is, however, not related to an absolute seasonal advance, but to the advantage of arriving at the breeding area before ‘competitors’ [20, 21]. Consequently, natural selection favours individuals within the same species or population that migrate at a faster pace under comparable conditions, as even a few days earlier arrival can yield significant reproductive benefits. Since investing in higher migration speed proves advantageous across species and migration strategies in spring [30, 32], we assume that the differences between migration strategies found in autumn are also present in spring.

Another migratory decision is in which direction to start the migratory flight from stopover [33]. While departure direction is a key feature in orientation and navigation research [33], fewer studies have included this trait in an ecological perspective of migration strategies [34, 35]. As departing in a seasonally inappropriate direction results in detours, time-constrained trans-Saharan migrant birds may have a stronger urge to more precisely depart towards the assumed migratory destination than pre-Saharan migrants.

To fill parts of these knowledge gaps, we studied the departure decisions and behaviour of trans- and pre-Saharan songbird migrants in a comparable approach at a stopover site during the spring migration, complementing a previous similar autumn study [17]. For this, we captured three trans-Saharan and two pre-Saharan migrant songbird species on Helgoland, a small island in the German Bight. By using a regional network of radio-receiving stations covering large parts of the German Bight (Fig S2), we automatically recorded their natural departure behaviour. By assuming comparable differences in the stopover decision making between pre-Saharan and trans-Saharan migrants in autumn [17], we specifically predict that pre-Saharan migrants in spring will (i) have longer minimum stopover duration, (ii) time their night-to-night departure decision more strongly in relation to daily meteorological conditions, and (iii) show later nocturnal departure timing than trans-Saharan migrants, independent of meteorological conditions. Furthermore, we predict for both migration strategies that (iv) energy stores estimated at capture are positively correlated with the departure probability and (v) with the nocturnal departure timing during the first night. Regarding the departure directions, we predict that (vi) trans-Saharan migrants set off from Helgoland towards the seasonally appropriate migratory direction, as derived from ring recoveries. Therefore, the mean departure direction might not differ from the mean direction of ring recoveries. Additionally, the angular variation, i.e. the concentration, of these directions might be similar. In contrast, we predict the opposite for pre-Saharan as their departure might be less oriented. To validate that these initial departure directions (first 1–10 km) are meaningfully maintained over larger distances (50–100 km), we predict (vii) that the birds do not adjust their flight direction after leaving Helgoland when crossing the North Sea towards the mainland.

## Materials and methods

### Study site and species

**Fig. 1:**
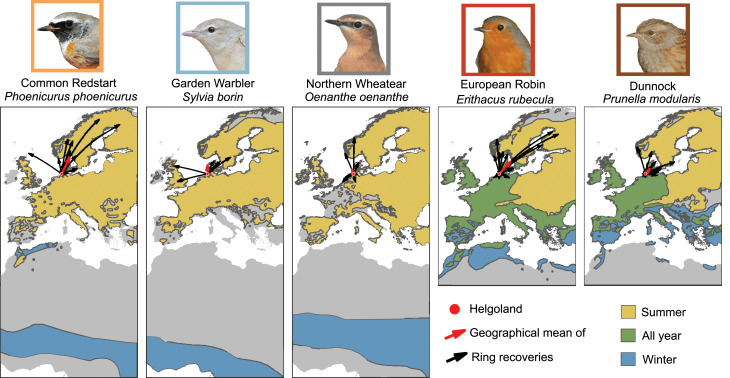
Distribution maps and ring recoveries of the study species. Portraits of species are given above their corresponding, restricted distribution maps (provided by Birdlife [51]). ‘All year’-occurrence (green) does not necessarily mean that the European Robin and Dunnock are resident in these areas but that some breeding birds migrate south-west in the autumn and their ‘breeding areas’ are filled by conspecifics from more northerly populations during winter. Black arrows indicate ring recoveries from birds caught on Helgoland (red dot) during spring migration (March-May) and were re-sighted in the same year until the end of the breeding season (end June). The red arrow indicates the geographical mean location of the ring recoveries, see Fig. S1 for more details. Bird photos: TKa.

The study was carried out on Helgoland (54°11′ N, 07°53′ E), a small island (ca. 2 km^2^) in the North Sea about 50 km off the German coast (Fig. 1). The extent of the ecological barrier to be crossed varies significantly with the departure location and the intended area of arrival, ranging from 50 km (German coast) to 800 km (Scotland) or even 1700 km (Iceland) [34, 36]. Average flight duration for songbirds to the closest mainland at the German coast is about 1–1.5 h [37, 38], which is substantially less than during an average migratory endurance flight of 8–10 h [39, 40]. Migrants that leave Helgoland and do not interrupt their flight when reaching the coast have resumed migration, whereas birds stopping over along the coast have probably performed landscape movements [41, 42] in search of more favourable stopover areas [3]. Under deteriorating weather conditions (upcoming rain, fog or strong headwinds, e.g. >10 m/s) Helgoland attracts many migratory landbirds that seek a landing place in the North Sea [34]. Songbirds stopover on Helgoland between a few hours to several weeks. The duration depends among others on the species, body condition, age, sex, season, weather, food availability, competition, and predator danger [34]. Songbirds often use the island for fuelling, resting and recovering [43, 44]. Generally, Helgoland seems to be used opportunistically as a stopover site [36], which is indicated for instance by the extremely low recapture rate, i.e. across seasons, of birds ringed on the island [45].

The focal species of the study are three trans-Saharan migrants, namely Common Redstarts (*Phoenicurus phoenicurus*; Redstarts hereafter), Garden Warblers (*Sylvia borin*) and Northern Wheatears (*Oenanthe oenanthe*; Wheatears hereafter; both subspecies *leucorhoa* and *oenanthe*, see supplemental for more information), and two pre-Saharan migrant species, namely European Robins (*Erithacus rubecula*; Robins hereafter) and Dunnocks (*Prunella modularis*). None of the study species regularly breeds on the island [36], so that all individuals of this study were on migration. Except the Dunnock, the species are night-time migrants. All species are broad front migrants crossing, in our case, the German Bight (Fig. S2) presumably on their way towards their Scandinavian breeding grounds. The *leucorhoa* Wheatears breed on Iceland, Greenland or Eastern Canada. As the neighbouring landmasses of Helgoland falls within the potential breeding range of all species (Fig. 1), we do not know the exact breeding areas and hence their remaining migration distances. Ring recoveries suggest that at least some songbirds passing Helgoland breed as far north as in northern Finland and Sweden (Fig. 1).

### Field procedures

Birds were trapped using mealworm-baited spring traps, funnel traps or mist nets. Birds were caught between 07:00–18:00 (local time) between mid-March and end of May 2017 and 2018 (in 2017 only Robins and Wheatears), the main migration period of the species during spring migration [36]. The following parameters, among others, were routinely taken for each bird: muscle size after [46] on a scale from “0” (sternum sharp, muscles depressed) to “3” (sternum yet distinguishable, muscles slightly rounded), body mass (± 0.1 g) with an electronic balance and maximum wing length to the nearest 0.5 mm [47]. The latter was used as a measure of body size [48].

After ringing, each bird was fitted with a coded radio-tag (NTQB-2 Avian Nano Tag; 0.29 g; burst intervals varied between 1.9 and 5.9 s; Lotek Wireless Inc., Newmarket, ON, Canada) using a leg-loop harness individually adjusted to the bird’s body size [49]. Mass of radio tags (including harness < 0.35 g) did not exceed 3% of the individual’s body mass (Redstart: max = 2.8%; Wheatear: max = 1.8%; Garden Warbler: max = 2.3%; Robin: max = 2.4%; Dunnock: max = 2.1%) [50]. To obtain an unbiased natural sample, no birds were specifically selected for tagging.

### Tracking of departure events

An automated digital radio-telemetry system was used to determine the birds’ departure events (timing and direction) on Helgoland. The system consists of four SensorGnome receivers (https://sensorgnome.org) located at three sites and equipped with a total of 12 radially aligned Yagi antennas (6EL Yagi antennas; Vårgårda Radio AB, Sweden) [52]. Furthermore, radio-telemetry stations were established at the German coastline (Fig. S2). This telemetry system continuously recorded radio signals on a utilised frequency (here 150.1 MHz, which is free for animal tracking in Germany, Federal Network Agency) to determine the exact timing of individual departure events on Helgoland and also to track individuals within the region, i.e. the German Bight [52]. This system is part of the Motus Wildlife Tracking System, see https://motus.org [53, 54].

Departures from Helgoland as recorded by this system are generally characterised by a rapid increase in signal strength detected by all/most antennas (bird takes off from the ground), followed by a decrease in signal strength from a decreasing number of antennas to signal loss (bird leaves the site in a certain direction) [17, 37, 52]. All tracking data were inspected visually. If the specific departure pattern described above was missing, we did not determine departure time. This was the case in 1 of the 47 Redstarts, 4 of the 44 Garden Warblers, 10 of the 58 Wheatears, 39 of the 81 Robins and 9 of the 56 Dunnock radio-tagged for this study. Since we could not exclude that these birds were caught by a predator during stopover or that their radio-transmitters dropped or stopped transmitting (technical failure), they were omitted from all analyses. As we analysed nocturnal departure decisions, we excluded birds that left Helgoland during daytime. It is not known whether these diurnal departures are only landscape movements or true departures to resume migration [42]. Daytime departure was recorded in 3 out of 46 Redstarts, in 1 out of 40 Garden Warblers, in 6 out of 48 Wheatears, in 3 out of 42 Robins, and in 4 out of 47 Dunnocks.

The bird’s nocturnal departure timing was defined as the time of highest signal strength during each departure event (for more details see [17]; Fig. S2). Based on this timing, we calculated the respective temporal difference between initial capture and departure (minimum stopover duration in days), the binary departure decisions of each bird for each day/night they stayed on Helgoland (stayed vs. departed), as well as the bird’s nocturnal departure timing in relation to night length (proportion of night at departure). For the latter, a description as a proportion of night was needed to compare all species, as they passed the study site at different periods in spring and thus experienced different night durations and sunset/sunrise times. Additionally, we calculated the degree of the sun´s elevation at departure because it is an important physical trait with effects on orientation cues [55]. To estimate the departure direction of the bird, we calculated a weighted circular mean of the directions in which the receiving antennas were oriented (see [17]).

After the birds had left Helgoland, some passed the radio-receivers along the German Bight (Fig. S2). We calculated the geographical bearing between the site of the first arrival on the coast and Helgoland to analyse the directional consistency of the flight.

### Estimating energy stores

We estimated the bird’s energy stores following the approach detailed in Kelsey et al. [56], by applying species- and muscle-score-specific equations to estimate the bird’s lean body mass (see supplemental material). All individuals had a muscle score of “2.”

### Weather data

To describe the wind conditions of individual birds during their stopover and at the time of their departure on Helgoland, we used the airspeed function (EQ^Airspeed^) of the R-Package RNCEP (NOAA; Boulder, CO, USA; https://psl.noaa.gov/data/gridded/data.ncep.reanalysis.html; spatial resolution: 2,5 × 2,5°; temporal resolution: 6 h; [57, 58]). With that function, we calculated the flow assistance [m/s] towards the assumed departure direction derived from ring recoveries (Fig. 1), assuming a pressure level of 1000 mbar (close to surface) and the assumed airspeed of the birds. Airspeed is the speed of a bird relative to the surrounding air though which the bird is flying [7]. This resulted in the following values for Redstarts: expected direction towards the breeding area = 18° and airspeed = 13 m/s, for Garden Warblers: 0° and 13 m/s, for Wheatears: 347° and 13 m/s, for Robins: 31° and 11 m/s, and for Dunnocks: 35° and 13 m/s, respectively.

Further meteorological data was provided by the German Weather Service (automated weather station on Helgoland; DWD; ftp://ftp-cdc.dwd.de/pub/CDC/observations_germany/climate/hourly/). These measurements (air pressure [mbar], air temperature [°C] and cloud cover [x/8]) were assigned to the time of sunset for each day a bird was on Helgoland as well as to the individual nocturnal departure time. Furthermore, we calculated the change in air pressure and air temperature as the difference between the last measurement before sunset and/or departure and the corresponding measurement 24 h before departure time.

### Statistical analyses

All statistical analyses were performed with the software R version 4.3.2 [59] and are available in the supplemental R-Script with the corresponding supplemental data. All continuous explanatory variables were centred to zero and scaled to one standard deviation (z-transformed) at the species level before modelling. We checked multicollinearity among the continuous explanatory variables using a variance inflation factor (VIF) and sequentially dropped the covariate with the highest VIF until all VIF < 2 [60]. Visual inspection of standard diagnostic plots did not show serious deviation from model assumptions in either of the models.

#### (i) Minimum stopover duration

To assess whether the minimum stopover durations differed between the species, we applied a Poisson regression model (generalised linear mixed model) with species (categorical, five levels: Redstart, Garden Warbler, Wheatear, Robin and Dunnock) as explanatory variable and an observation-level random term as a random factor to correct for overdispersion [61].

#### (ii) Meteorological conditions – night-to-night departure probability

We assessed the effect of meteorological conditions on night-to-night departure probability using time-dependent Cox proportional hazards models implemented in the ‘survival’ package [62]; for more information about the Cox proportional hazards model see Packmor et al. [17]. We estimated the departure probability as a function of species (fixed variable), day of year (time-varying variable), and a set of meteorological variables (time-varying variables). Meteorological variables included in the initial model were flow assistance (continuous), cloud cover (proportional, in eighths), change [sunset to sunset, 24 h] in atmospheric pressure (Δ atmospheric pressure; continuous), and change [sunset to sunset, 24 h] in air temperature (Δ air temperature; continuous). Additionally, the initial model included the two-way interactions between species and each of the other variables. On three days during stopover and on three departure days of six Robins and one day during stopover of a Dunnock, crosswinds were higher than the species-specific airspeed so that we could not calculate flow assistance for those days [58]. Therefore, we had to exclude those seven of the of 883 datapoints from all Cox proportional hazards models that contain meteorological variables. We tested the influence of these seven removed datasets by assuming slightly higher airspeeds (Robin: 12.5 instead of 11 m/s; Dunnock: 14.3 m/s instead of 13 m/s) of the species and found no influence on the results (see R-Code).

We applied an automated model selection using the ‘dredge’ function implemented in the R-package ‘MuMIn’ [63]. The subsequent model averaging was conducted for all models with a ΔAICc < 2 [64], for which we used the ‘model.avg’ function implemented in the R-package ‘MuMIn’. We provide average estimates and corresponding 95% confidence intervals for all independent variables that remained in the selected models with a ΔAICc < 2; estimates are averages over the models where the parameter appeared. Variables included in the selected models after model averaging are detailed in Tab. S1.

#### (iii) Meteorological conditions – nocturnal departure timing

We assessed the effects of species and meteorological variables on nocturnal departure timing (proportion of night at departure) by fitting beta regression models using the ‘betareg’ function implemented in the ‘betareg’ package [65]. The model included birds leaving Helgoland during the first or any other night following capture. The initial model included species, day of year (1 January = 1; continuous), flow assistance, cloud cover, Δ atmospheric pressure, and the two-way interactions between species and each of the variables. The variable Δ air temperature was excluded due to collinearity with VIF > 2. Afterwards we performed automated model selection similar to the analysis in “(ii) Meteorological conditions – night-to-night departure probability” (see paragraph above). Variables included in the models are detailed in Tab. S2.

#### (iv) Energy stores – departure probability first night

The effect of energy stores on departure probability was restricted to the first night following capture, because all energy store estimates were based on the birds’ body masses at capture. As this estimate becomes less reliable with each day of stopover [42], we restricted this (iv) and the following analysis (v) to the first night following capture. Furthermore, we had to exclude the Dunnock from those models because only one individual departed in the first night after capture.

To assess how variation in energy stores affected departure probability during the first night following capture, we fitted a binary logistic regression model. The initial model included energy stores (continuous), species (categorical: four levels: Redstart, Garden Warbler, Wheatear and Robin), day of year (1 January = 1; continuous) and the two-way interaction between energy store and species as explanatory variables. The two-way interaction was not significant (*p* > 0.05) and had to be removed from the initial model before re-running the final model [66].

#### (v) Energy stores – nocturnal departure timing first night

We assessed the effect of energy stores on nocturnal departure timing (proportion of night at departure) of birds that left Helgoland during the first night following capture by fitting beta regression models using the ‘betareg’ function implemented in the ‘betareg’ package [65]. The initial model included energy stores, species, day of year, the two-way interaction between energy stores with species and day of year. The two-way interaction between energy stores with species was not significant (*p* > 0.05) and had to be removed from the initial model before re-running the final model [66].

#### (vi) Departure direction

To assess whether birds set off from Helgoland towards the seasonally appropriate migratory direction, we compared their direction with the directions of ring recoveries from birds previously caught on Helgoland during spring migration (March-May) and were re-sighted in the same year until the end of the breeding season (end June). To test for directionality, we applied the Rayleigh test and calculated the confidence interval of the mean direction with the ‘mle.vonmises.bootstrap.ci’-function with 10,000 iterations, both implemented in the R package ‘CircStats’ [67]. To test for differences in mean/median angle between radio-tracked departure directions and directions of ring recoveries, we used the P-test, implemented in R by [68]. To test for differences in angular variance, i.e. concentration, we used Levene´s test implemented in the R package ‘lawstat’ [69].

#### (vii) Directional consistency

To assess the directional consistency of the departure flight, we assessed the correlation between the departure direction from Helgoland and the bearing of the coastal arrival station with the circular correlation test imbedded in the R Package ‘CircStats’ [67]. We only included birds, which departed in a direction between 13° to 236° clockwise (north-northeast to southwest), as this is the geographical arrangement of the coastline around Helgoland that was covered with radio-receiving stations (Fig. S2). This was the case for 71 of 95 birds.

## Results

### (i) Minimum stopover duration

**Fig. 2:**
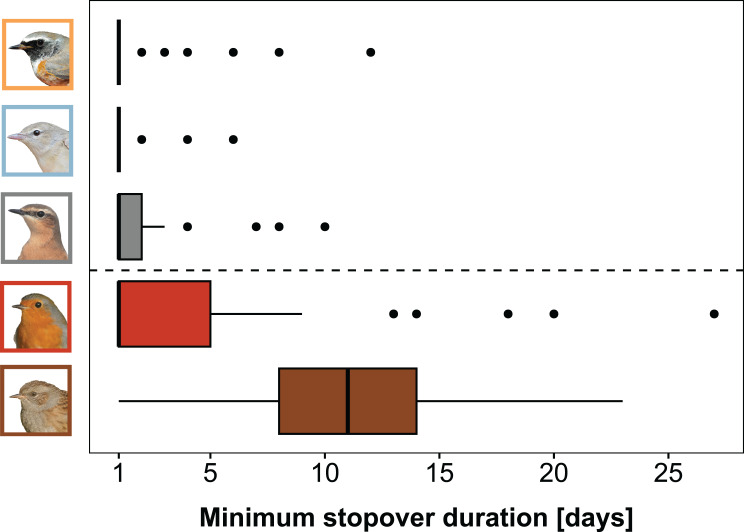
Prediction (i): Variation in minimum stopover duration. The three trans-Saharan songbird species (above dashed line from top to bottom: Redstart, Garden Warbler, Wheatear) show significantly shorter minimum stopover duration than the pre-Saharan migrants (below dashed line: Robin, Dunnock) during spring migration. Box plots show the 5th, 25th, 50th, 75th and 95th percentile as well as outliers (dots). Bird photos: TKa

Trans-Saharan migrants (median: 1 day) stayed 6 days less than pre-Saharan migrants (median: 7 days) at the stopover site. The species differed in their minimum stopover duration on Helgoland during spring (Poisson regression model: Intercept (Redstart): 0.37 (SE 0.16), *p* = 0.018; Garden Warbler: -0.26 (SE 0.23), *p* = 0.27); Wheatear: 0.11 (SE 0.22), *p* = 0.62; Robin: 0.77 (SE 0.21), *p* = 0.0002; Dunnock: 1.91 (SE 0.19), *p* < 0.0001; Fig. S3). The model revealed that the two pre-Saharan migrants, Robins (median = 1 days; range = 1–27 days; *n* = 39) and Dunnocks (median = 11 days; range = 1–23 days; *n* = 43), stayed significantly longer on the island than the trans-Saharan migrants, Redstarts (median = 1 day; range = 1–12 days; *n* = 43), Garden Warblers (median = 1 day; range = 1–6 days; *n* = 39) and Wheatears (median = 1 day; range = 1–10 days; *n* = 42; Fig. 2, Fig. S3).

### (ii) Meteorological conditions – night-to-night departure probability

**Fig. 3:**
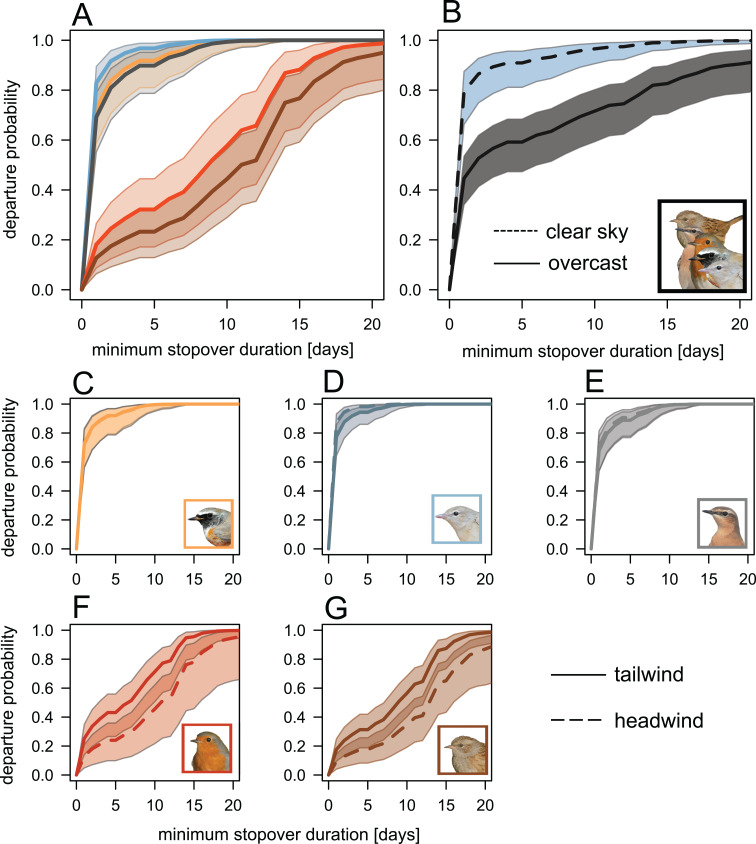
Prediction (ii): Night-to-night departure probability predicted by time-dependent Cox proportional hazards models. Predictions (lines) and associated 95% confidence intervals (shaded areas) are given. (**A**, **C-G**) Colour scheme corresponds to species: Redstart (yellow-orange), Garden Warbler (light blue), Wheatear (grey), Robin (red) and Dunnock (brown). (**A**) Species-specific differences in the night-to-night departure probability. (**B**) All species were combined to visualise the effect of cloud cover on the night-to-night departure probability. Minimum (broken line; clear sky) and maximum (solid line; overcast) of the scaled cloud cover birds experienced at sunset were used. (**C-G**) Species-specific effect of wind condition on night-to-night departure probability, given for the 25th percentile (broken line; headwind) and 75th percentile (solid line; tailwind) of the scaled air speed flow assistance birds experienced at sunset. A plot depicting the effect of Julian day is provided in Fig. S4. Bird photos: TKa

Robins and Dunnocks showed a lower night-to-night departure probability than Redstarts, Garden Warblers and Wheatears (Table 1; Fig. 3A). Cloud cover had a significant negative effect on the night-to-night departure probability in all species (Table 1; Fig. 3B). The significant two-way interaction between species and flow assistance indicate that the night-to-night departure probability of the two pre-Saharan migrants (Robin and Dunnock), but not of the three trans-Saharan Migrants (Redstart, Garden Warbler and Wheatear), was affected by flow assistance with higher departure probability under tailwind than under headwind conditions (Table 1; Fig. 3C-G). Day of year had a positive effect on departure probability for Robins with higher departure probability early in the season (Table 1; Fig. S4).

**Table 1:** Prediction (ii): effects of meteorological conditions and day of year on night-to-night departure probability. Average model estimates, adjusted standard errors (SE), 95% confidence intervals (CIs) and associated *p*-values of parameters included in the candidate time-dependent Cox proportional hazards models (see Tab. S1). *P*-values < 0.05 are given in bold font. Reference category for species is Redstart

Parameter	Estimate ± SE	95% CI	*p*
Species (Garden Warbler)	0.328 ± 0.224	-0.11–0.766	0.143
Species (Wheatear)	-0.082 ± 0.211	-0.495–0.331	0.698
Species (Robin)	-1.887 ± 0.316	-2.506 - -1.267	**< 0.001**
Species (Dunnock)	-2.282 ± 0.291	-2.853 - -1.711	**< 0.001**
Cloud cover	-0.346 ± 0.116	-0.572 - -0.119	**0.003**
Day of year	0.101 ± 0.16	-0.212–0.414	0.527
Flow assistance	0.004 ± 0.179	-0.347–0.355	0.981
Δ atmospheric pressure	-0.018 ± 0.05	-0.237–0.069	0.721
Δ air temperature	0.017 ± 0.05	-0.073–0.241	0.730
Flow assistance x Species (Garden Warbler)	-0.340 ± 0.303	-0.934–0.254	0.262
Flow assistance x Species (Wheatear)	-0.111 ± 0.237	-0.576–0.355	0.641
Flow assistance x Species (Robin)	0.658 ± 0.274	0.122–1.195	**0.016**
Flow assistance x Species (Dunnock)	0.658 ± 0.264	0.141–1.175	**0.013**
Cloud cover x Species (Garden Warbler)	0.039 ± 0.15	-0.367–0.74	0.793
Cloud cover x Species (Wheatear)	0.005 ± 0.105	-0.42–0.469	0.960
Cloud cover x Species (Robin)	-0.052 ± 0.147	-0.702–0.207	0.722
Cloud cover x Species (Dunnock)	-0.084 ± 0.19	-0.814–0.015	0.657
Day of year x Species (Garden Warbler)	-0.554 ± 0.257	-1.059 - -0.050	**0.031**
Day of year x Species (Wheatear)	-0.067 ± 0.216	-0.490–0.356	0.757
Day of year x Species (Robin)	-1.099 ± 0.276	-1.64 - -0.558	**< 0.001**
Day of year x Species (Dunnock)	-0.354 ± 0.227	-0.800–0.091	0.119

### (iii) Meteorological conditions – Nocturnal departure timing

**Fig. 4:**
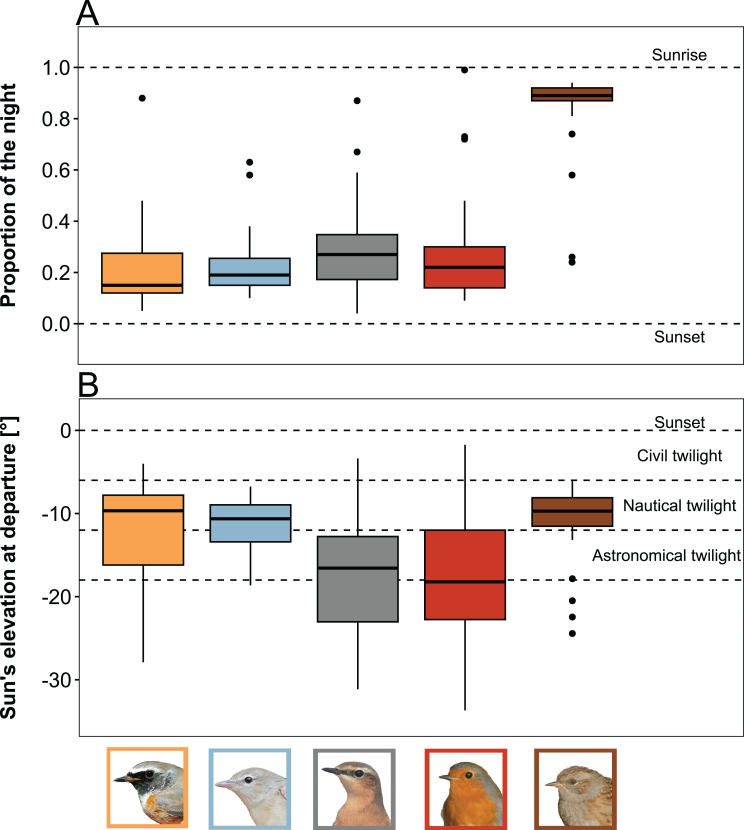
Prediction (iii): Variation in nocturnal departure timing as observed in five songbird species during spring migration. Box plots show the 5th, 25th, 50th, 75th and 95th percentile as well as outliers (dots). Species displayed are, from left to right: Redstart, Garden Warbler, Wheatear, Robin and Dunnock. (**A**) Nocturnal departure timing is expressed as proportion of night at departure. (**B**) Nocturnal departure timing is expressed as sun´s elevation at departure. A figure depicting the nocturnal departure time in minutes after sunset can be found in Fig. S8. Bird photos: TKa

Nocturnal departure timing, measured as the proportion of night at departure, differed between the species. Redstarts (median = 0.15; range = 0.05–0.88; *n* = 43) departed earlier within the night than Wheatears (median = 0.27; range = 0.04–0.87; *n* = 42) and Robins (median = 0.22; range = 0.09–0.99; *n* = 39), while Dunnocks departed later than the other species (median = 0.89; range = 0.24–0.94; *n* = 43; Table 2; Fig. 4A; Fig. S5). We found no effect of the meteorological conditions on the nocturnal departure timing, but several significant interactions between species and day of year. (Table 2, Fig. S6). The majority of all birds (73%) departed during nautical and astronomical twilight, i.e. at sun elevations between − 6 and − 18° (Fig. 4B; Fig. S7). Whereas Dunnocks departed during the morning twilight, all other species depart during evening twilight (Fig. 4; Fig. S7).

**Table 2:** Prediction (iii): effects of weather variables and day of year on nocturnal departure timing (proportion of night at departure). Average model estimates of beta regression models after automatic model selection, adjusted standard errors (SE), 95% confidence intervals (CIs) and associated *p*-values of parameters included in the candidate models in Tab. S2 are shown. *P*-values < 0.05 are given in bold font. Reference category for species is Redstart

Parameter	Estimate ± SE	95% CI	*p*
Intercept	-1.225 ± 0.116	-1.454 - -0.997	**< 0.001**
Species (Garden Warbler)	0.070 ± 0.164	-0.255–0.394	0.674
Species (Northern Wheatear)	0.358 ± 0.158	0.046–0.670	**0.024**
Species (European Robin)	0.326 ± 0.162	0.007–0.646	**0.045**
Species (Dunnock)	2.861 ± 0.174	2.518–3.204	**< 0.001**
Cloud cover	0.038 ± 0.091	-0.148–0.242	0.679
Day of year	-0.311 ± 0.112	-0.532 - -0.090	**0.006**
Δ air temperature	0.006 ± 0.025	-0.059–0.148	0.803
Cloud cover x Garden Warbler	0.042 ± 0.113	-0.207–0.439	0.712
Cloud cover x Northern Wheatear	0.013 ± 0.097	-0.276–0.347	0.896
Cloud cover x European Robin	0.144 ± 0.215	0.074–0.721	0.503
Cloud cover x Dunnock	-0.029 ± 0.111	-0.421–0.261	0.795
Day of year x Garden Warbler	0.404 ± 0.164	0.080–0.727	**0.014**
Day of year x Northern Wheatear	0.124 ± 0.158	-0.187–0.435	0.435
Day of year x European Robin	0.535 ± 0.162	0.216–0.854	**0.001**
Day of year x Dunnock	0.132 ± 0.169	-0.202–0.465	0.438

### (iv) Energy stores – night-to-night departure probability first night

Departure probability during the first night was positively correlated with energy stores in all species, meaning that birds with more fat stores were more likely to continue migration in the following night (Table 3; note that the Dunnock was excluded from this analysis, see methods). As the interaction between species and energy stored was not significant and therefore removed from the final model (see methods), we do not find a species-specific pattern (see also Fig. S9).

**Table 3:** Prediction (iv): effects of energy stores and day of year on departure probability during the first night following capture. Model estimates, adjusted standard errors (SE), 95% confidence intervals (CIs) and associated *p*-values of parameters are shown. *P*-values < 0.05 are given in bold font. Reference category for species is Redstart. Note Dunnocks were excluded for this model, see methods

Parameter	Estimate ± SE	95% CI	*p*
Intercept	1.266 ± 0.371	0.577–2.046	**0.001**
Energy stores	0.530 ± 0.202	0.150–0.945	**0.009**
Day of Year	-0.282 ± 0.186	-0.658–0.074	0.128
Species (Garden Warbler)	0.559 ± 0.584	-0.566–1.757	0.338
Species (Wheatear)	-0.738 ± 0.496	-1.735–0.222	0.137
Species (Robin)	-0.869 ± 0.501	-1.878–0.100	0.083

### (v) Energy stores - nocturnal departure timing first night

**Fig. 5:**
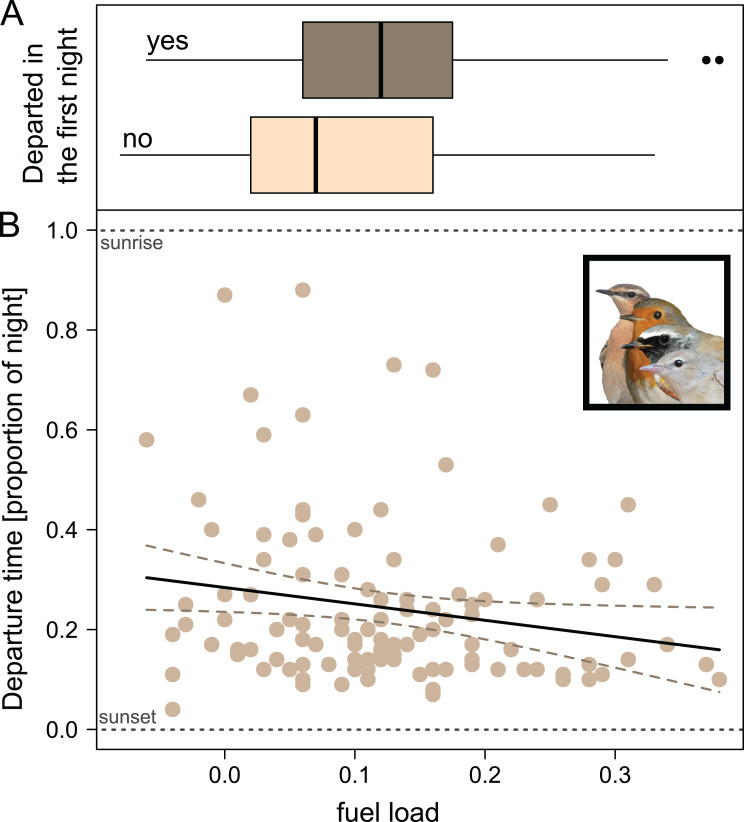
Prediction (iv)+(v): Energy stores influencing songbirds´ departures. Effect of fuel load on (**A**) departure decision and (**B**) on departure time within the night of birds departing in the first night after radio tagging. Data are aggregated over the species Redstarts, Garden Warblers, Wheatears and Robins. Dunnocks were excluded as only one bird departed in the first night. Bird photos: TKa

Nocturnal departure timing during the first night was negatively correlated with the birds’ energy stores with higher energy stores being associated with earlier departures (Table 4; Fig. 5). The significant two-way interaction between species and day of year demonstrated that Redstarts and Wheatears advanced nocturnal departure timing with seasonal progress, while this was not the case in the Garden Warblers and Robins (Table 4, Fig. S6), similar to what we found with the complete dataset in Prediction (iii).

**Table 4:** Prediction (v): effect of energy stores and day of year on nocturnal departure timing (proportion of night at departure) during the first night following capture. Estimates, standard errors (SE), 95% confidence intervals (CIs) and associated *p*-values of all parameters included in the final beta regression model are shown. *P*-values < 0.05 are given in bold font. Reference category for species is Redstart. Note that Dunnocks were excluded for this model, see methods

Parameter	Estimate ± SE	95% CI	*p*
Intercept	-1.221 ± 0.128	-1.471 - -0.970	**< 0.001**
Energy stores	-0.138 ± 0.070	-0.274 - -0.002	**0.047**
Species (Garden Warbler)	0.085 ± 0.177	-0.261–0.432	0.629
Species (Wheatear)	0.356 ± 0.183	-0.003–0.715	0.052
Species (Robin)	0.231 ± 0.195	-0.152–0.614	0.237
Day of year	-0.337 ± 0.126	-0.583 - -0.091	**0.007**
Day of year x Species (Garden Warbler)	0.547 ± 0.212	0.131–0.964	**0.010**
Day of year x Species (Wheatear)	-0.077 ± 0.177	-0.424–0.271	0.665
Day of year x Species (European Robin)	0.242 ± 0.191	-0.132–0.616	0.204

### (vi) Departure direction

**Fig. 6:**
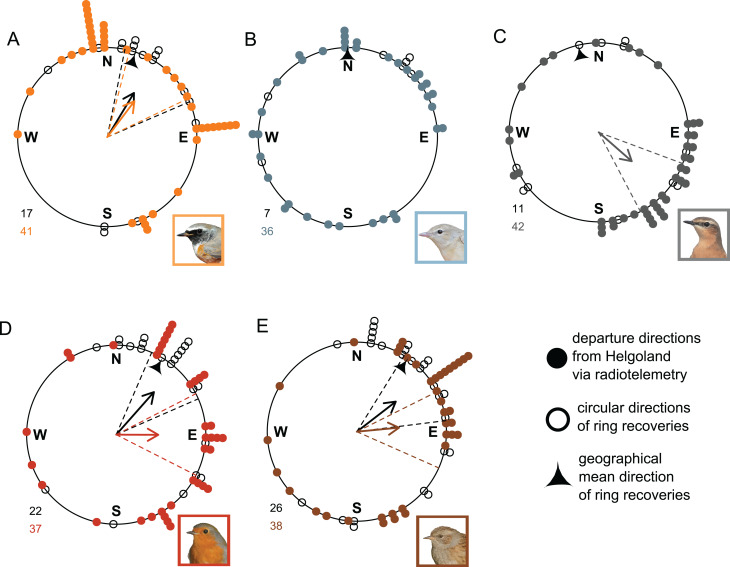
Prediction (vi): Directional decision from the stopover site. Migratory direction from Helgoland as derived from radio telemetry (filled coloured circles, coloured arrows and coloured dashed confidence intervals) and ring recoveries (open circles, black arrows and black dashed confidence intervals) from five songbird species: (**A**) Redstart, (**B**) Garden Warbler, (**C**) Wheatear, (**D**) Robin and (**E**) Dunnock. Arrows and their confidence intervals (dashed lines) are only shown for significantly oriented datasets. Correspondingly coloured numbers indicate sample size. Pointy triangle indicates geographical mean of ring recoveries, see Fig. 1 or Fig. S1 for a map. Bird photos: TKa

The mean departure directions obtained from radio tracking for Redstarts (37°; Rayleigh-test: *r* = 0.503, *p* < 0.001, *n* = 41), Robins (90°, Rayleigh-test: *r* = 0.470, *p* < 0.001, *n* = 37) and Dunnocks (85°, Rayleigh-test: *r* = 0.473, *p* < 0.001, *n* = 38) were not biologically meaningfully different from their mean spring migration direction based on ring recoveries (Redstarts: 31°, Rayleigh-test: *r* = 0.577, *p* = 0.002, *n* = 17); Robins (41°, Rayleigh-test: *r* = 0.635, *p* < 0.001, *n* = 22 and Dunnocks: 52°, Rayleigh-test: *r* = 0.550, *p* < 0.001, *n* = 26; Fig. 6). The mean radio-tracked departure direction of Wheatears was significantly oriented towards southeast (131°, Rayleigh-test: *r* = 0.490, *p* < 0.001, *n* = 42), whereas their directions of ring recoveries were not significantly oriented (Rayleigh-test: *r* = 0.073, *p* = 0.945, *n* = 11). In Garden Warblers, neither direction was significant (departure directions: Rayleigh-test: *r* = 0.205, *p* = 0.220, *n* = 36; ring recoveries: Rayleigh-test: *r* = 0.653, *p* = 0.061, *n* = 7; Fig. 6). Using P-test, we found significant differences in mean/median angle between radio-tracked departure and ring-recovery directions for Redstarts (*p* = 0.001), Robins (*p* < 0.001) and Dunnocks (*p* < 0.001), but not for Garden Warblers (*p* = 0.403) and Wheatears (*p* = 0.105). Using Levene’s test, we found significant differences in angular variance, i.e. concentration, between radio-tracked departure and ring-recovery directions for Redstarts (test statistic = 5.81, *p* = 0.019), but not for Garden Warblers (test statistic = 3.667, *p* = 0.062), Wheatears (test statistic = 2.92, *p* = 0.094), Robins (test statistic = 0.07, *p* = 0.791) and Dunnocks (test statistic = 0.01, *p* = 0.935).

### (vii) Directional consistency over the German bight

**Fig. 7:**
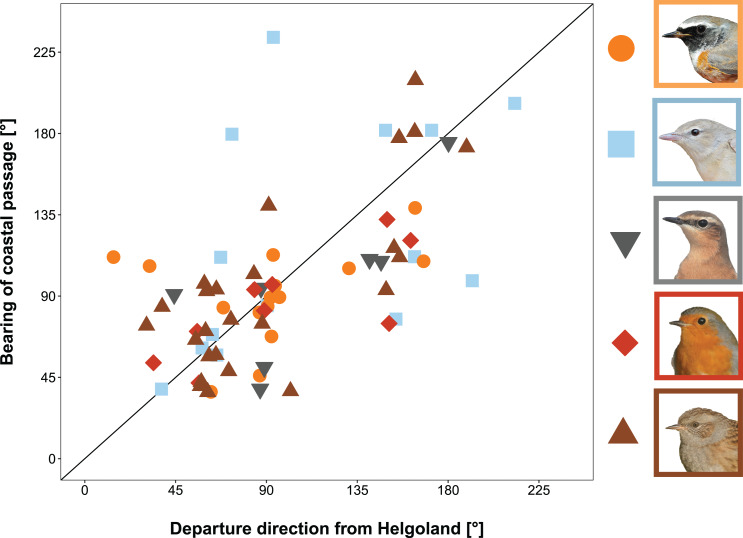
Prediction (vii): Directional consistency of migratory flights. Consistency was analysed by comparing the departure direction from Helgoland within the first ∼ 1–10 km with the bearing of the passage location on the coast after 50–100 km for five migrant songbirds, Redstart (orange circle), Garden Warbler (blue square), Wheatear (grey pointed downward triangle), Robin (red rhombus), and Dunnock (brown pointed upward triangle). The black diagonal line marks the 1:1 ratio, meaning that the bird did not change its flight direction. Points are slightly (< 5°) jittered to prevent overlap. For geographical map of the German bight see Fig. S2. Bird photos: TKa

Across species, the coastal sites that were passed during the migratory endurance flights after leaving Helgoland were in line with the initial departure direction from the island (circular correlation: *r* = 0.632, test statistic = 4.76, *p* = < 0.001, *n* = 71; Fig. 7). This means that the majority of birds did not change their initially chosen departure direction from Helgoland (first 1–10 km) over a distance of 50–100 km. The correlation remained significant, even when birds, which departed from Helgoland outside of the sector covered with receiving stations along the coast (see methods; Fig. S2), were included (*r* = 0.514, test statistic = 4.52, *p* = < 0.001, *n* = 95), although these birds bias the dataset towards inconsistent flight directions. Species-specific correlations were not significant, except for Dunnock, the species with the highest sample size in this analysis (Redstart: *r* = 0.330, test statistic = 1.18, *p* = 0.238, *n* = 16; Garden warbler: *r* = 0.481, test statistic = 1.78, *p* = 0.075, *n* = 14; Wheatear: *r* = 0.701, test statistic = 1.71, *p* = 0.087, *n* = 7; Robin: *r* = 0.744, test statistic = 1.84, *p* = 0.067, *n* = 9; Dunnock: *r* = 0.732, test statistic = 3.15, *p* = 0.002, *n* = 25).

## Discussion

Our study demonstrates that stopover departure decisions differ between pre-Saharan and trans-Saharan migrants during the spring migration in a comparable way to autumn migration [17]. In both migratory seasons, pre-Saharan migrants are less time-constrained and appear to minimise the energy costs of migration more than trans-Saharan migrants, which seem to have a higher urge to minimise the time spent during migration. This general pattern suggests that the decision-making process may be rigidly adapted according to the migration strategy and associated time constraints. Season-specific selection pressure seems to be superimposed on the general pattern and favour faster migration in spring than in autumn. Despite that adaptation, the pronounced difference in the stopover departure decisions between pre-Saharan and trans-Saharan migrants as documented during autumn migration [17] remains similarly pronounced during spring migration.

### Predictions (i)-(iii): differences between species

The overall speed of migration depends more on the total stopover duration than on the travel speed during the migratory endurance flight, so shortening the stopover duration would be an effective proximate mechanism to accelerate spring migration [8, 32, 58, 70]. Pre-Saharan migrants stayed longer on Helgoland than trans-Saharan migrants (Figs. 2 and 3), which is indicative for the generally slower migration speed migration of the former than the latter. Thus, the two pre-Saharan migrants seem to have a generally lower urge to maximise the migration speed than the trans-Saharan migrants. Our results, therefore, suggest that the time constraints associated with the migration strategy still lead to generally different stopover departure decisions between the strategies. It is important to consider that the evolutionary advantage of a fast spring migration is to arrive at the breeding area before the ‘conspecifics’ and not before individuals of other species [20, 21].

While we find a species difference on the day-to day basis of stopover duration between pre- and trans-Saharan migrants, we did not find a clear pattern regarding the within-the-night departure decision (Table 4; Fig. 3). In general, most individuals set off in the first third of the night except the Dunnock which initiated its migratory flight during dawn [6] (Fig. 3). Interestingly, the majority of birds of all species, including the Dunnock, departed during nautical and astronomical twilight (Fig. 4). There is evidence that migratory songbirds use multiple compasses (geomagnetic field, stars, the sun, and polarised light patterns) to determine their migratory direction [33, 71, 72]. As the corresponding compass cues are accessible within that time window, this might indicate that this period is important for the orientation and navigation processes, potentially involving calibration of compass systems [33, 55, 73, 74].

### Predicitions (ii)-(iii): departure decisions and weather conditions

Wind, especially the proportion of tail-/head wind, is widely considered to be an essential determinant of the migratory behaviour of birds, as it affects current local flight conditions and the related energy costs of transport [75, 76]. It also gives information on the flight conditions expected in the near future [75, 76]. Our results show that the departure decision, i.e. departure probability, of the two pre-Saharan migrants was positively affected by flow assistance (Fig. 3). Analogously, in another study, Black Redstarts (*Phoenicurus ochruros*), a pre-Saharan migrant, also selected nights with favourable winds for departure [77], and therefore tend to follow the energy-saving strategy, cf [78]. For the three trans-Saharan migrants, we could not find such an effect. This is consistent with the optimal migration theory, which suggests that trans-Saharan migrants are less selective for favourable winds and are more likely to follow a time-minimiser strategy [17, 78–81]. As in autumn [17], flow assistance affects less strongly the stopover departure decision in trans-Saharan than in pre-Saharan migrants and the former seem to be more prone to invest the increased energy costs of flying in head- and crosswinds than the latter. Our results, therefore, supports our prediction (ii) that trans-Saharan would time their night-to-night departure decision less strictly in relation to wind conditions than pre-Saharan migrants.

Increasing cloud cover had a consistent negative effect on night-to-night departure decisions for all five species (Table 1). Thus, birds were more likely to depart under clear skies than under overcast conditions, which is in line with former studies [17, 82–86], but see [87, 88]. Two possible, non-mutually exclusive causes may explain this pattern. First, migrants delay their departure if the conditions for nocturnal orientation and navigation are not optimal, e.g. the star compass is not visible [33, 83, 85, 89, 90]. Second, overcast conditions are usually associated with an increased probability of precipitation, which in turn poses a hazard during flight especially for landbirds crossing large bodies of water [91]. We argue that cloud cover generally affects departure decision regardless of the migration strategy because of its consequences for the bird’s orientation and navigation abilities and survival probability [17, 33].

In contrast to the night-to-night departure decisions, we found no effect of weather variables on nocturnal departure timing (Table 2). The length of the night determines the overall speed of the migration [2, 92], as it fundamentally constrains the maximum flight distance feasible within a given night, cf [40]. The four exclusive night-time migrants (Redstart, Garden Warbler, Wheatear and Robin) resumed migration shortly after sunset. Since the decision to resume migration is probably made several hours before sunset [93], the actual departure, in terms of the exact timing, is probably initiated as soon the environmental conditions are favourable to maximise the nightly migration distance and thus overall travel speed [2]. The same may apply to Dunnocks, but for dawn and not dusk, as they migrate into the day (Fig. 4) [6].

### Predicitions (iv) + (v): departure decisions and energy stores

As this study did not identify the reasons why the migrants had landed on Helgoland, we do not know what functions the stopover site was supposed to fulfil for the birds, cf [3]. However, we speculate that lean birds probably interrupted their migratory endurance flight for accumulating energy stores, reviewed in [42], while birds with higher energy stores probably landed on the island for other reasons, e.g. avoiding unfavourable weather conditions [94] or recovering from the exhausting migratory endurance flight [43], cf [3]. The fact that energy stores were positively correlated with the departure probability on the first night after capture (Table 3; Fig. 4) suggests that the birds with high energy stores did not require additional fuelling on the island and thus landed for reasons other than energy accumulation. This seems likely in our case because Helgoland has a high daily turn-over rate of migratory songbirds during migration and because of the high catching effort suggesting that many migrants are captured on their first day on the island, cf [95].

### Prediction (vi): departure direction

Both pre-Saharan species and one of three trans-Saharan species departed from Helgoland towards biologically comparable directions as derived from their species-specific ring recoveries (Fig. 6). Therefore, we do not find support for the prediction that the direction of migratory flights of trans-Saharan migrants are better aligned with the seasonally appropriate direction, or that they are less variable than those of pre-Saharan migrants. Why Wheatears left Helgoland in a south-easterly direction and Garden Warblers in no preferred direction remains elusive. Similar observations, where birds did not depart in their seasonally appropriate migratory direction were made with four songbird species in North America [35]. It might be that these individuals either overshot their breeding area and had to correct for that or that they originated from more geographically different breeding areas, cf. [96], than individuals of the other species. The directional consistency (Prediction vii) supports this conclusion.

Our radio-tracking data (Fig. 6) shows that the departure direction of individual birds from a stopover site on a regional scale can, but does not have to correspond to the seasonally appropriate migration direction as derived from other methods. These include for instance, Emlen funnels [97], ring recoveries on a larger geographical scale (Fig. 1), radar tracking of migrating birds in horizontal flights [98, 99] or tracking full migration [100] trajectories. However, the limitations of any method used to derive a ‘seasonally appropriate direction’ must be thoroughly considered. For example, ring recoveries, as used in this study, depend heavily on the spatial and temporal recapture/-sighting probabilities. Thus, their detection probability varies significantly in space and time due to local avian research activities, such as the location of bird ringing stations, the population density of people reporting such re-sightings, and the spatial distribution of land masses and oceans.

Brown & Taylor [101] showed in a larger geographical scale with radio telemetry that nocturnal songbird migrants do not necessarily migrate in a series of successive flights, all oriented in the seasonally appropriate direction. Instead, successive flights might be ‘undirected’ within a region, even though they ultimately head to the migratory destination. This may be especially the case, when the stopover site does not fulfil the function for which the migrants interrupted their migratory flight [3], birds might enhance ‘undirected’ regional movements to find a more suitable stopover site. Only when stopover needs are met do birds usually resume migration in the seasonally appropriate direction. Understanding the function of stopovers and the subsequent consequences for departure direction is also crucial for orientation and navigation research, where local departures are often used as a directional proxy for the migratory direction [33].

### Directional consistency

Songbirds that resume migration from Helgoland seem to have decided at least upon departure in which direction to continue migration (Fig. 7). This is in line with orientation/navigation studies on Helgoland, where Robins (*n* = 202) and Wheatears (*n* = 100) showed significantly consistent flight directions after leaving the island (mean deviation < 22°) [37, 38, 102]. These results are in slight contrast to Sjöberg and Nilsson study [98] which showed larger variation in vanishing bearing of radio-tracking migratory songbirds leaving Falsterbo in southern Sweden during autumn than the true travel directions tracked by radar. We propose that the birds in the studies from Helgoland either did not need to adjust their migratory flight direction during the ascend on cruising altitudes or that they reached their cruising altitudes already within the surveyed area of the receiving stations on Helgoland.

## Conclusions

This study shows that pre-Saharan migrants differ from trans-Saharan migrants in their stopover departure decisions during spring migration, and that their behavioural difference in the decision is comparable to autumn migration [17]. It, therefore, appears to be a general pattern that pre-Saharan migrants are less time-constrained and have a greater urge to migrate in an energy-saving strategy than trans-Saharan migrants. At the same time, it is unclear whether the results of this study are geographically generalisable because individual behavioural response to specific intrinsic and extrinsic factors may vary both along the migration route and over the seasonal period [52, 95].

The low selectivity for environmental conditions among trans-Saharan migrants may indicate low flexibility or a limited reaction norm to such changes. This could potentially expose trans-Saharan migrants to higher risks of global change, which in turn may partly explain why trans-Saharan migrants suffer more severe population declines than pre-Saharan migrants [103]. Pre-Saharan migrants, with their longer migration period and apparently more flexible reaction norm to environmental conditions in terms of stopover departure decisions, may have more potential to adjust or adapt to global change than trans-Saharan migrants.

## Electronic supplementary material

Below is the link to the electronic supplementary material.

Supplementary Material 1

## Data Availability

The supplementary material including all data tables and Software Code is available in the Oldenburg research data repository [DARE] under following address: https://doi.org/10.57782/WXZE6H
