# Supplementary material for: Stopover departure decisions in spring: pre-Saharan migrants stay longer and are more selective for favourable wind than trans-Saharan migrants
Source: Mov Ecol. 2025 Sep 22;13:64. doi: 10.1186/s40462-025-00575-0 (PMC12455787; doi:10.1186/s40462-025-00575-0)
Supplement: Supplementary file 1 — Supplementary Material 1 [file 40462_2025_575_MOESM1_ESM.pdf]

Supplemental material to:

Stopover departure decisions in spring: pre-Saharan migrants stay longer and are more selective for favourable wind than trans-Saharan migrants

Thomas Klinner*,[1,2], Thiemo Karwinkel*,[#,1,3], Florian Packmor[1,4] & **Heiko Schmaljohann[1,3]**

[1] Institute of Avian Research "Vogelwarte Helgoland", An der Vogelwarte 21, 26386 Wilhelmshaven, Germany
[2] Naturwacht Brandenburg, Heinrich-Mann-Allee 18/19, 14473 Potsdam, Germany
[3] Carl von Ossietzky Universität Oldenburg, School of Mathematics and Science, Institute of Biology and Environmental Sciences, Ammerländer Heerstraße 114–118, 26129 Oldenburg, Germany
[4] Lower Saxon Wadden Sea National Park Authority, Virchowstraße 1, 26382 Wilhelmshaven, Germany

*shared first authors
#author of correspondence: thiemo.karwinkel@uni-oldenburg.de

This Material contains supplementary figures, tables and methods.
Data tables and Software Code is available in the Oldenburg research data repository [DARE] under following address: https://doi.org/10.57782/WXZE6H.

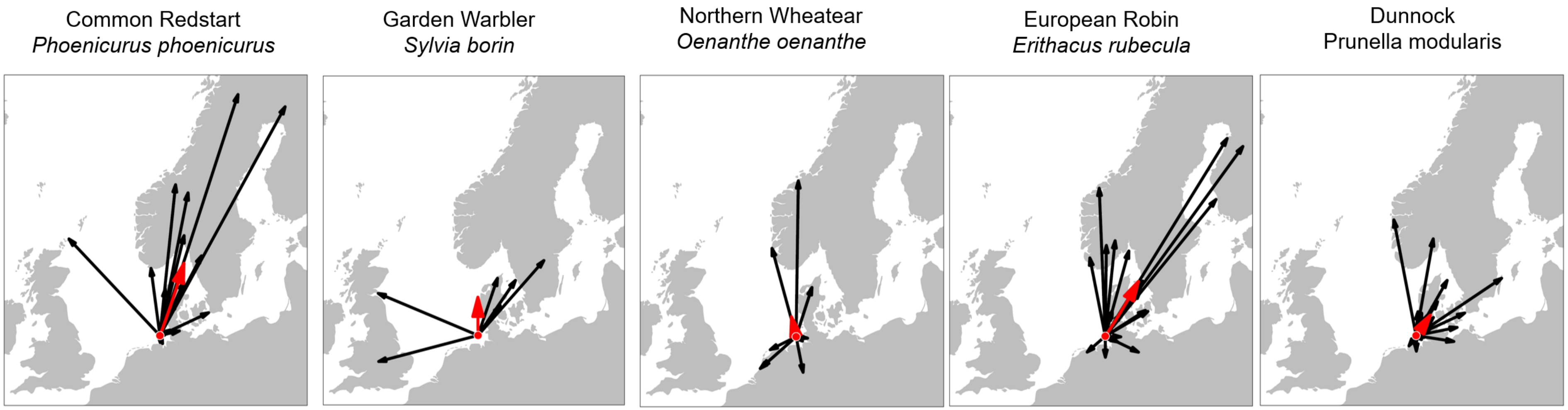

Fig. S1. Ring recoveries of the study species. Black arrows indicate ring recoveries from birds caught on Helgoland (red dot) during spring migration (March-May) and re-sighted in the same year until the end of the breeding season (end June). The red arrow indicates the geographical mean location of the corresponding ring recoveries.

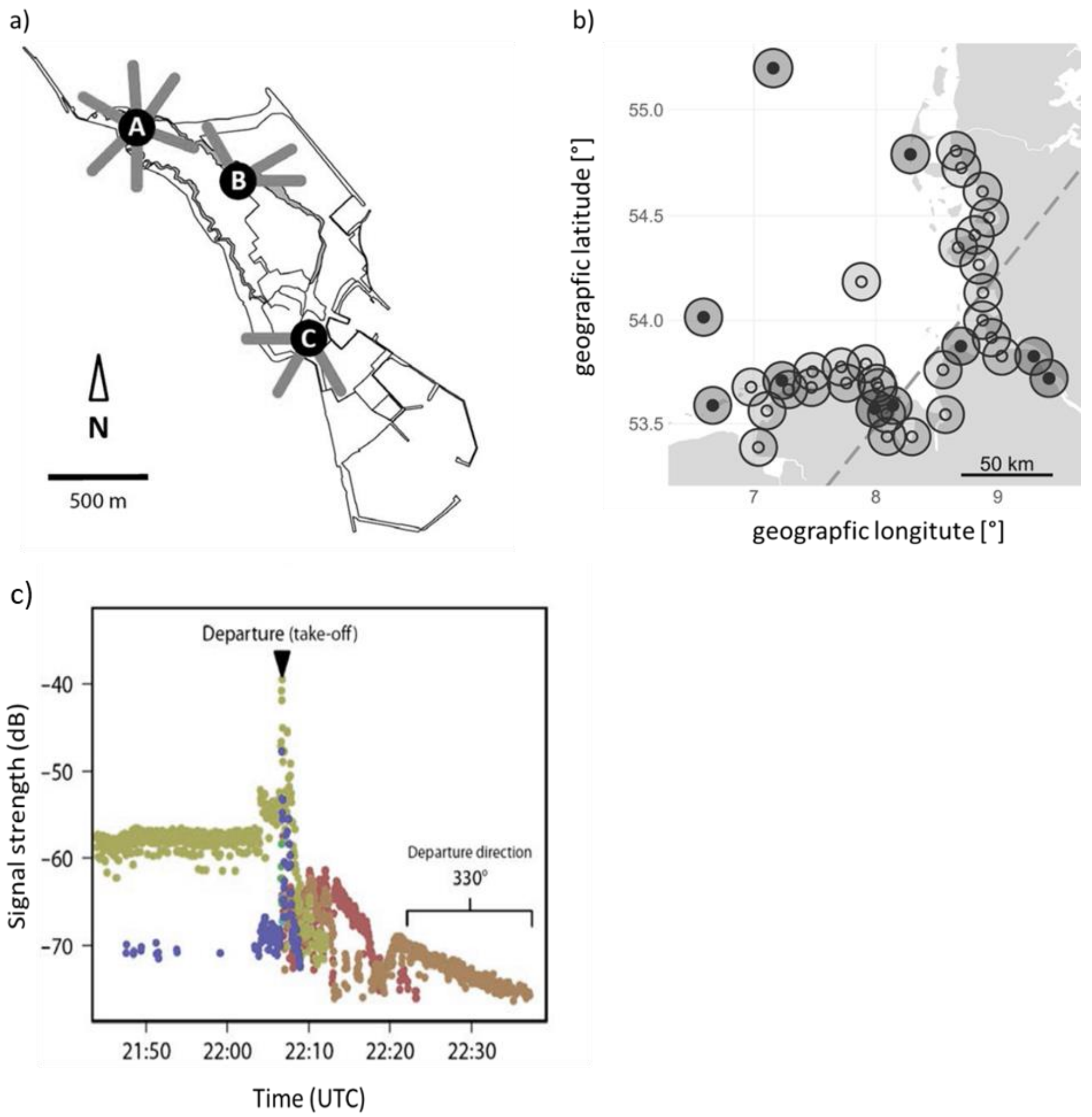

Fig. S2. a) Radio-telemetry system on Helgoland including 12 antennas at three radio-receiving stations (A, B, C) and its corresponding directions (after Müller et al. 2018). b) Location of automated radio-telemetry stations at the German North Sea coast (after Brust et al. 2019). c) Example of a departure event of a bird leaving Helgoland (after Müller et al. 2018). A rapid increase in signal strengths reflects the take-off, while the last signals indicate the departure direction.

Wheatear subspecies

For this comparative study between pre- and trans-Saharan songbird migrants, we pooled the data of both wheatear subspecies, namely *oenanthe* and *leucorhoa* on the species level. We are aware that differences in the stopover ecology between these subspecies exist (see; Brust et al 2022, Schmaljohann et al. 2016, Müller et al. 2018), but solely focus on species (group) differences for this preditcion.

To be sure that the subspecies did not behave differently, we also tested whether they differed in the main migration characteristics, on which the predictions were based upon. The probability of departing or not departing in the first night after tagging did not differ (chi-squared test: $X^2<0.001$, df=1, p=1). The stopover duration did not differ between the subspecies (Wilcoxon rank sum test: W=216, p=0.832). The departure time within the night, measured as minutes after sunset, did not differ (Wilcoxon rank sum test: W=236, p=0.781), which is in line with the study by Müller et al. (2018), who also tested Wheatears on Helgoland. The departure direction between the subspecies did not differ (Watson-wheeler test: W00.016, df=2, p=0.992).

Formulas for estimating fuel load for every species

First, we calculated the bird´s lean body mass after Kelsey et al. 2019.

for each individual Redstart$_i$ with muscle score “2”:

$$lean\ body\ mass_{Redstart_i,\ muscle\ score\ 2}[g]=6.69\ [g]+0.08\ \left[\frac{g}{mm}\right]*wing\ length_i\ [mm] \quad (1)$$

for each individual Garden Warbler$_i$ with muscle score “2”:

$$lean\ body\ mass_{G.\ Warbler_i,\ muscle\ score\ 2}[g]=13.1[g]+0.05\ \left[\frac{g}{mm}\right]*wing\ length_i[mm] \quad (2)$$

for each individual Wheatear$_i$ with muscle score “2”:

$$lean\ body\ mass_{Wheatear_i,\ muscle\ score\ 2}[g]=-11.07[g]+0.34\ \left[\frac{g}{mm}\right]*wing\ length_i[mm] \quad (3)$$

for each individual Robin$_i$ with muscle score “2”:

$$lean\ body\ mass_{Robin_i,\ muscle\ score\ 2}[g]=2.48\ [g]+0.17\ \left[\frac{g}{mm}\right]*wing\ length_i[mm] \quad (4)$$

for each individual Dunnock$_i$ with muscle score “2”:

$$lean\ body\ mass_{Dunnock_i,\ muscle\ score\ 2}[g]=1.41[g]+0.24\ \left[\frac{g}{mm}\right]*wing\ length_i[mm] \quad (5)$$

Second, we calculated the bird’s energy stores at capture as:

$$Evening\ energy\ stores_i=\frac{(body\ mass_i\ [g]-lean\ body\ mass_i\ [g])}{lean\ body\ mass_i\ [g]}$$

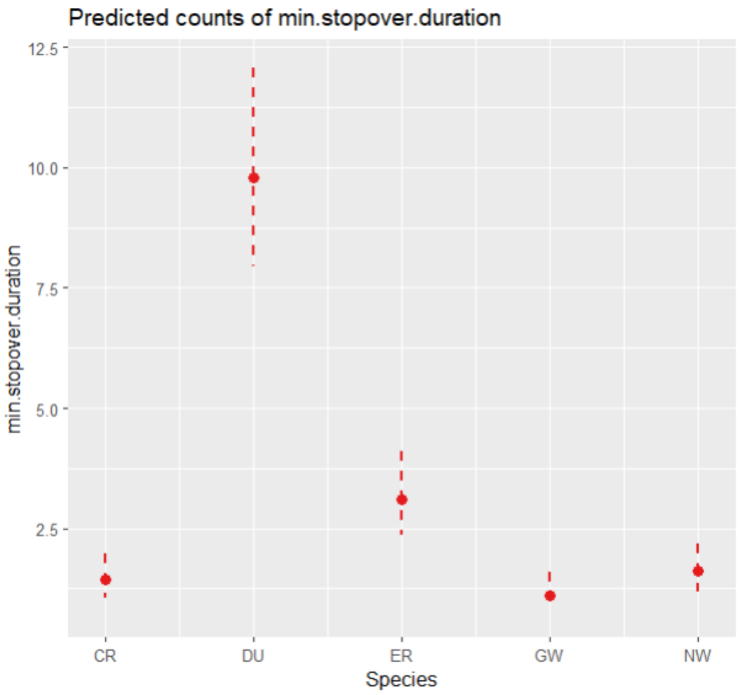

Fig. S3. Plotted marginal effects with 95% confidence intervals of the regression model testing the effects of species (CR=Common Redstart, DU=Dunnock, ER=European Robin, GW=Garden Warbler, NW=Northern Wheatear) on minimum stopover duration (min.stopver.duration) from prediction (i), see Results-section.

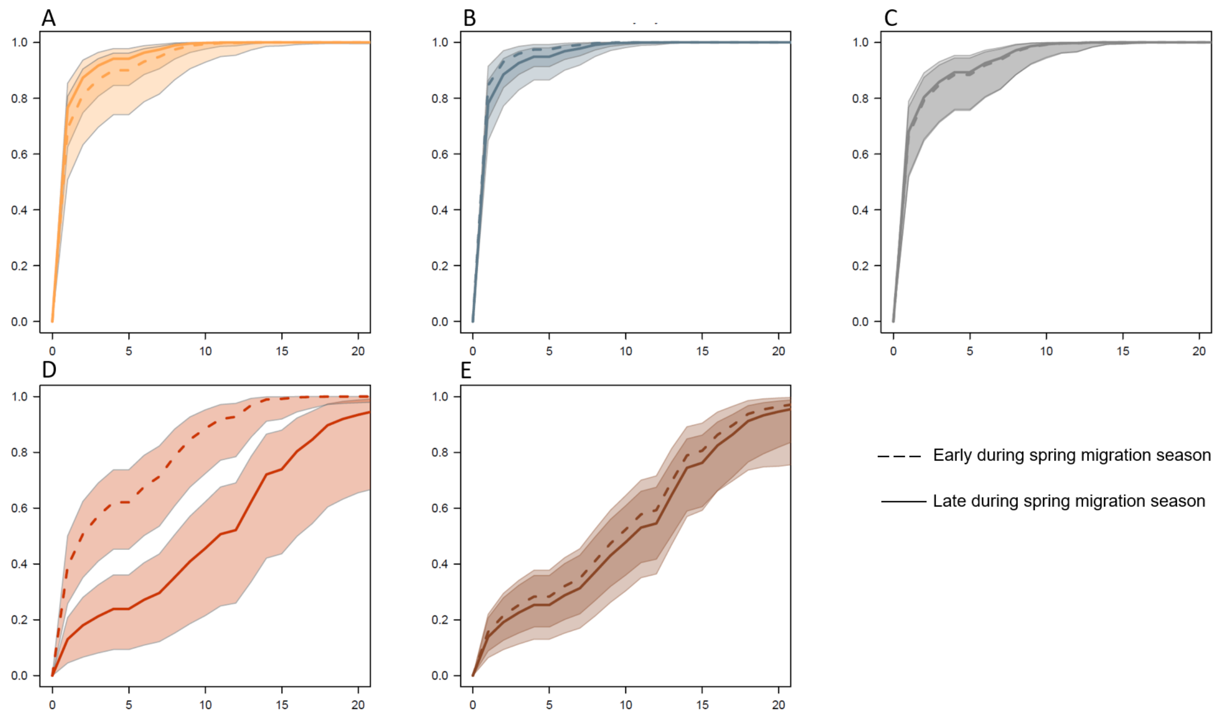

Fig. S4. Night-to-night departure probability predicted by time-dependent Cox proportional hazards models. Predictions (lines) and associated 95 % confidence intervals (shaded areas) are given for **(A)** Redstart (orange) **(B)** Garden Warbler (light blue), **(C)** Wheatear (grey), **(D)** Robin (red) and **(E)** Dunnock (brown). Species-specific effect of day of year on night-to-night departure probability, given for the 25th percentile (broken line; low day of year= early in the season) and 75th percentile (solid line; high day of year= late in the season) of the species-specifically scaled day of year.

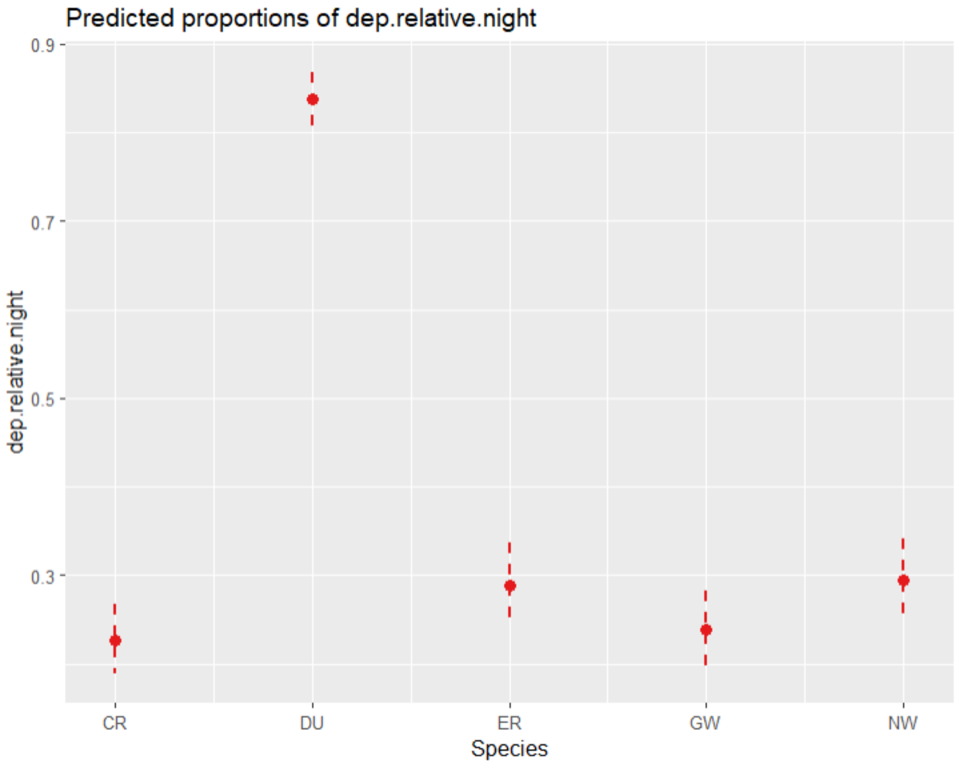

Fig. S5. Plotted marginal effects with 95% confidence intervals of the beta regression model testing the effects of species (CR=Commmon Redstart, DU=Dunnock, ER=European Robin, GW=Garden Warbler, NW=Northern Wheatear) on departure within the night (dep.relative.night) from prediction (iii), see table 1.

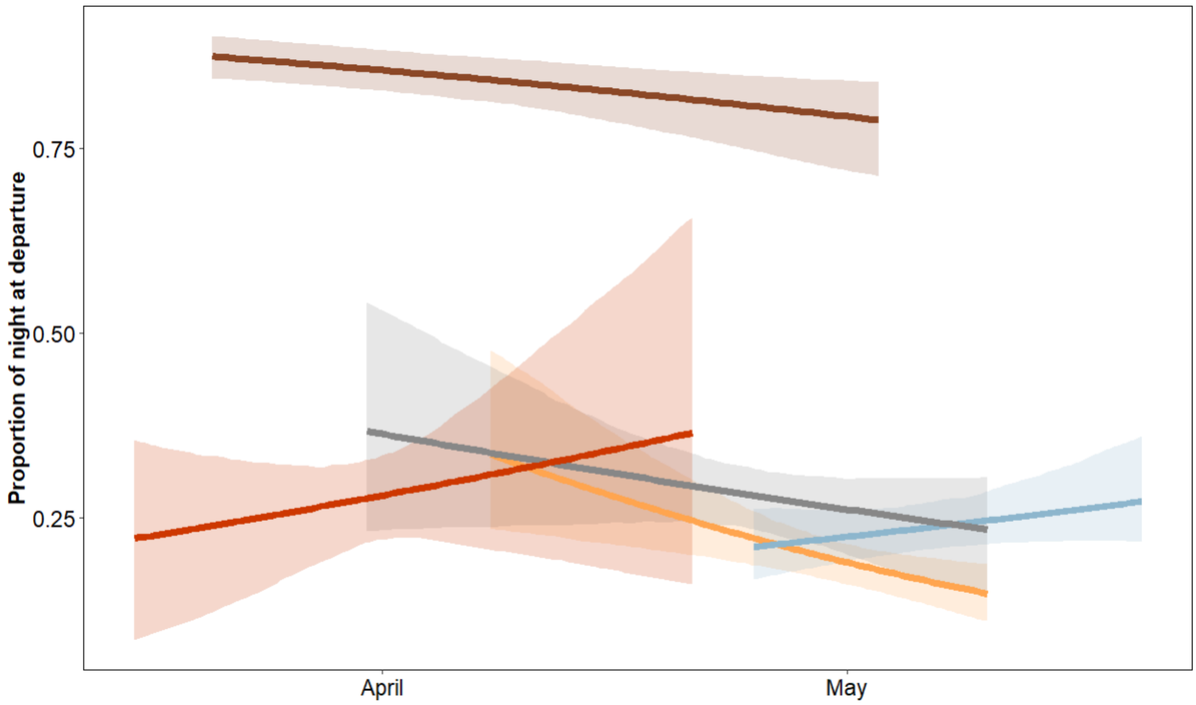

Fig. S6. Nocturnal departure timing over day of year for Redstarts (yellow-orange), Garden Warblers (lightblue), Wheatears (grey), Robins (red), and Dunnock (brown). Species-specific predictions (lines) and associated 95 % confidence intervals (shaded areas) are given.

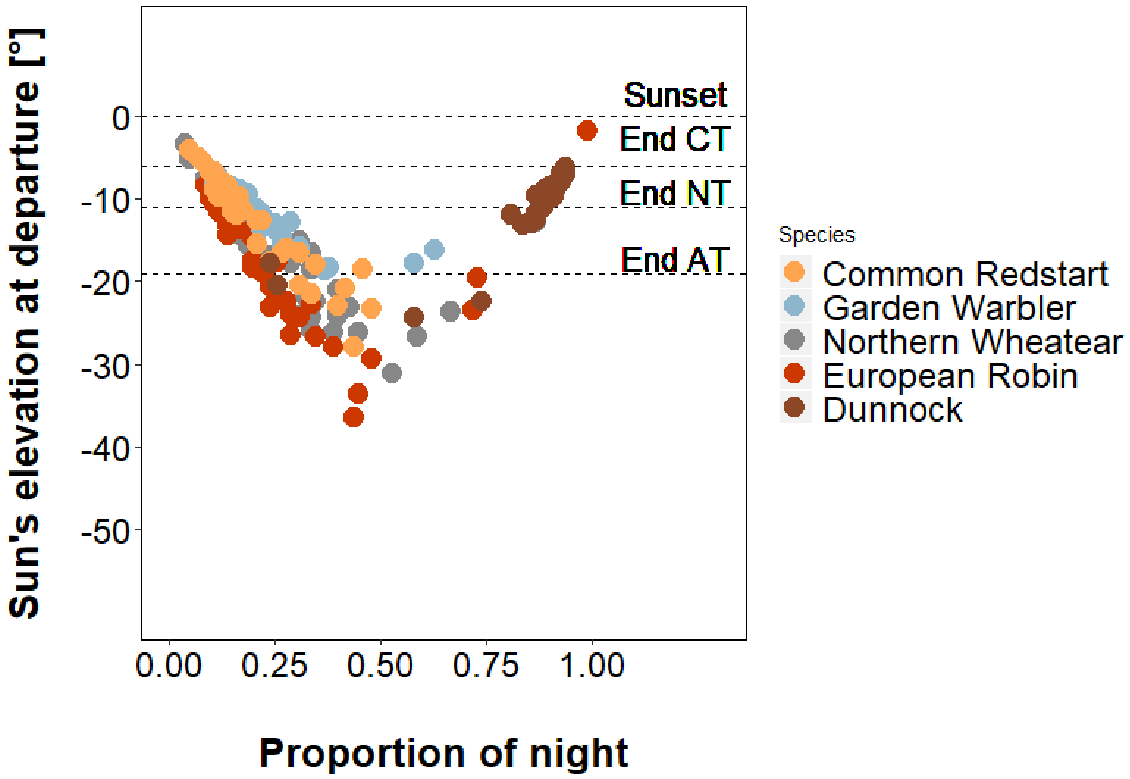

Fig. S7. Nocturnal departure time in 5 different songbird species during spring migration from Helgoland, as plotted on the sun´s elevation during the course of the night.

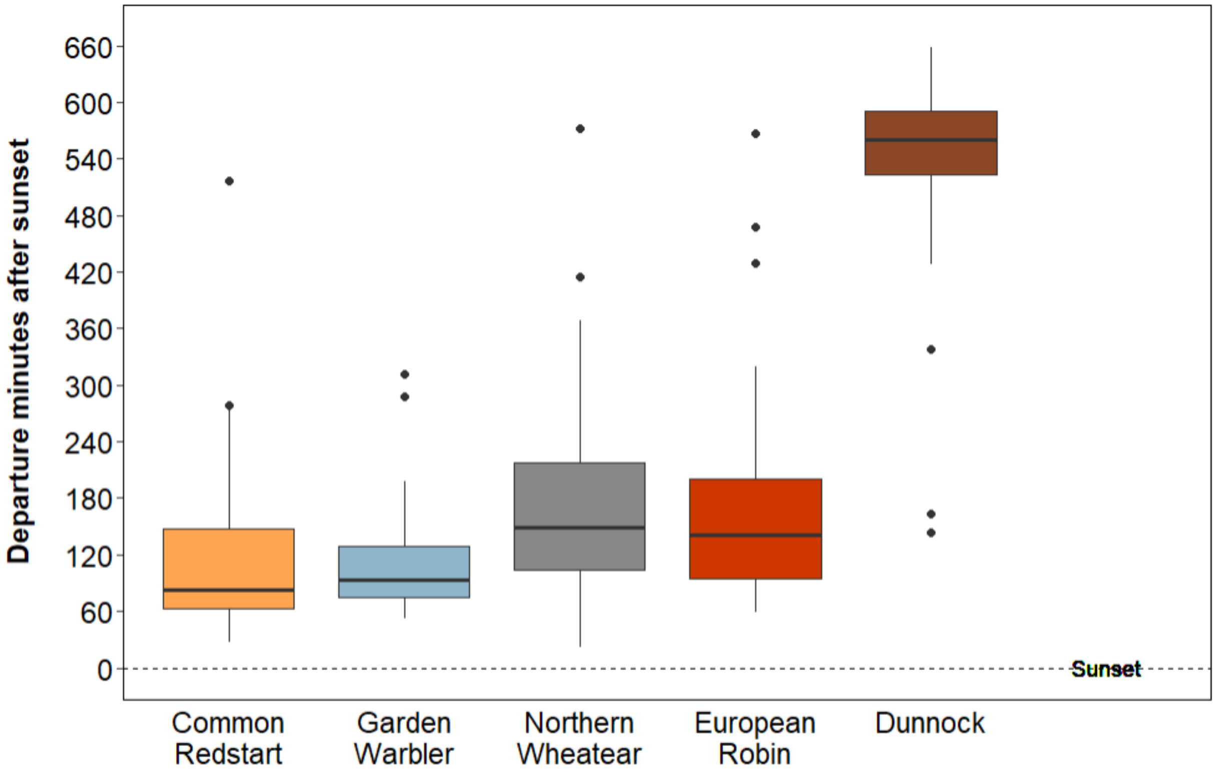

Fig. S8. Nocturnal departure time in five different songbird species during spring migration from Helgoland, as displayed in “minutes after sunset”.

Redstart: mean: 118 min, median: 83 min, 25th-75th percentile: 63-148 min, range: 27-516 min, n=43;

Garden Warbler: mean: 111 min, median: 93min, 25th-75th percentile: 75-129 min; range: 52-311 min, n=39;

Wheatear: mean: 174 min, median: 149 min; 25th-75th percentile: 104-218 min, range: 22-572 min, n=42;

Robin: mean: 174 min, median: 141 min, 25th-75th percentile: 95-201 min; range: 59-567 min, n=39;

Dunnock: mean: 538 min, median: 560 min, 25th-75th percentile: 524-591 min, range: 144-658 min, n=43

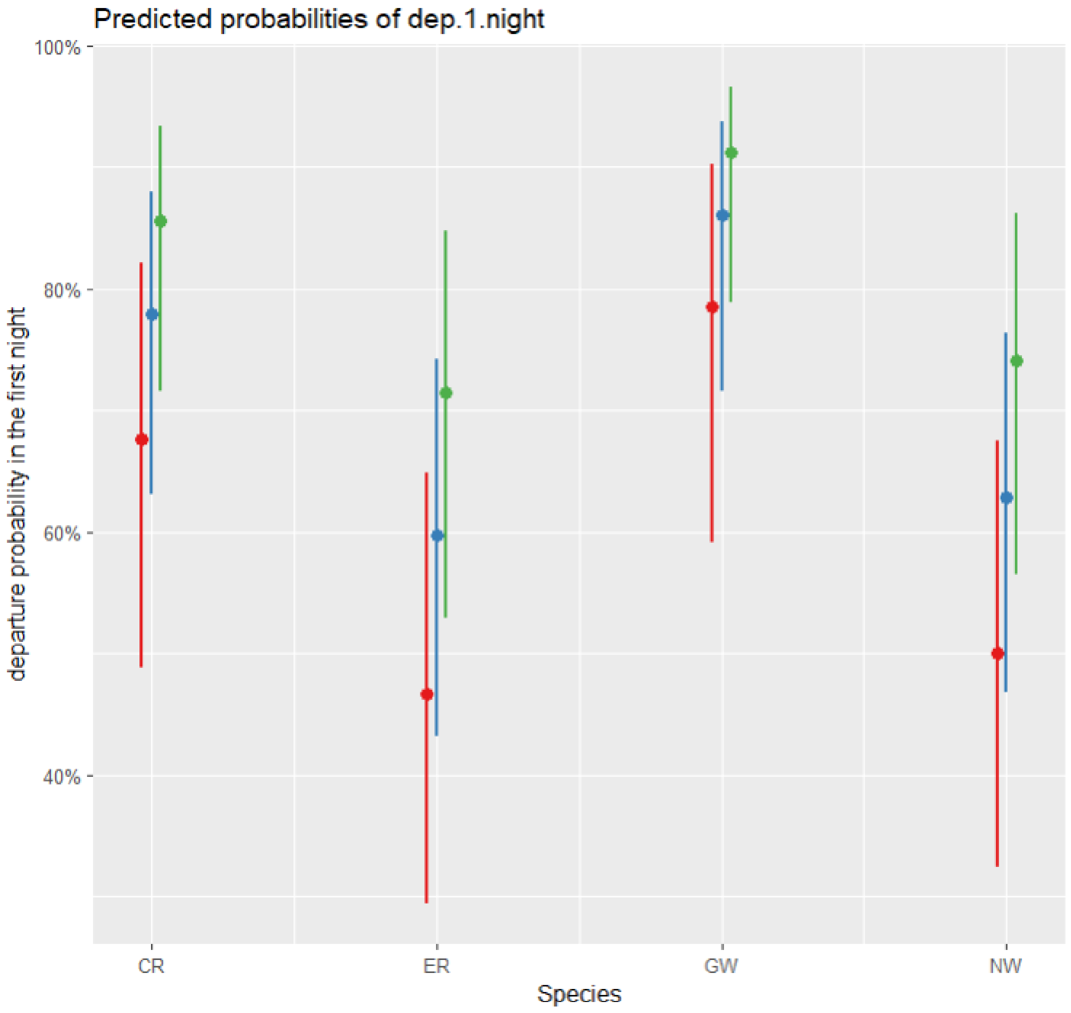

Fig. S9. Plotted marginal effects with 95% confidence intervals of the generalised linear model testing the effects of species (CR=Common Redstart, ER=European Robin, GW=Garden Warbler, NW=Northern Wheatear) and fuel load on departure probability in the first night after release for prediction (iv), see Tab. 3. Red lines show low, blue show median and green show high fuel load values. Dunnocks are not included in this analysis as energy stores can only be considered, when birds depart in the first night after catching, but only one Dunnock departed on the first night.

Tab. S1 Comparison of candidate time-dependent Cox proportional hazards models to assess the effects of weather variables on birds' departure probability (night-to-night departure decision from prediction (ii)). Models' coefficients and presence of factors are given. Degrees of freedom (df), second-order Akaike's information criterion values (AIC_c), AIC_c differences (Δ_i) and AIC_c weights (ω_i).

Model	Species	Flow assistance	Δ atmospheric pressure	Δ air temperature	Cloud cover	Day of year	Species x flow assitance	Species x cloud cover	Species x day of year	df	AICc	Δi AICc	ωi
1	+	-0.002			-0.3795	0.08901	+		+	15	1771.8	0	0.248
2	+	0.001			-0.2393	0.1132	+	+	+	19	1773	1.16	0.139
3	+	-0.014	-0.08449		-0.3541	0.1081	+		+	16	1773	1.17	0.138
4	+	0.036		0.08418	-0.385	0.1026	+		+	16	1773.1	1.24	0.134

Tab. S2. Comparison of candidate beta regression models to assess the effects of weather variables on birds' nocturnal departure timing (proportion of night at departure; within-night departure decision; from prediction (iii)). Models' coefficients and presence of factors are given. Degrees of freedom (df), second-order Akaike's information criterion values (AIC_c), AIC_c differences (Δ_i) and AIC_c weights (ω_i).

Model	Species	Δ air temperature	Cloud cover	Day of year	Species x cloud cover	Species x day of year	df	AIC_c	Δ_i AIC_c	ω_i
1	+		-0.007124	-0.317	+	+	16	-241	0	0.104
2	+		0.0933	-0.3051		+	12	-240.7	0.37	0.086
3	+			-0.3126		+	11	-239.8	1.24	0.056
4	+	0.04489	0.08703	-0.3066		+	13	-239.1	1.9	0.04

References Supplemental

Brust, V., Schmaljohann, H., & Hüppop, O. (2023). Two subspecies of a songbird migrant optimise departure from a coastal stopover with regard to weather and the route lying ahead. *Journal of Avian Biology*, *2023*(1-2), e03004.

Müller, F., Eikenaar, C., Crysler, Z. J., Taylor, P. D., & Schmaljohann, H. (2018). Nocturnal departure timing in songbirds facing distinct migratory challenges. *Journal of Animal Ecology*, *87*(4), 1102-1115.

Schmaljohann, H., Meier, C., Arlt, D., Bairlein, F., van Oosten, H., Morbey, Y. E., ... & Eikenaar, C. (2016). Proximate causes of avian protandry differ between subspecies with contrasting migration challenges. *Behavioral Ecology*, *27*(1), 321-331.
